# Role of the admission muscle injury indicators in early coagulopathy, inflammation and acute kidney injury in patients with severe multiple injuries

**DOI:** 10.1186/s13017-025-00593-8

**Published:** 2025-03-07

**Authors:** Liuquan Mu, Haideng Song, Mengdi Jin, Kaige Li, Yushan Guo, Nan Jiang

**Affiliations:** 1 Department of Trauma Center, China-Japan Union Hospital of Jilin University, Changchun, 130033 China; 2 Department of Emergency, Cheeloo College of Medicine, Weihai Municipal Hospital, Shandong University, Weihai, 264200 China; 3 Department of Epidemiology and Biostatistics, School of Public Health, Jilin University, Changchun, 130021 China

**Keywords:** Multiple injuries, Muscle injury indicators, Myoglobin, Coagulopathy, SIRS, AKI

## Abstract

**Backgrounds:**

Coagulopathy, inflammation and organ failure are common complications in trauma patients. This study aimed to explore the possible role of muscle injury indicators in early coagulopathy, systemic inflammatory response syndrome (SIRS), and acute kidney injury (AKI) in patients with severe multiple trauma.

**Methods:**

A retrospective analysis was performed using trauma center patient data from 2020 to 2023. The incidence of coagulopathy, SIRS and AKI in patients with multiple injuries were assessed. The relationship between Myoglobin, creatine kinase (CK), lactate dehydrogenase (LDH) and trauma severity was investigated, and the influence of these three muscle injury indicators on patient adverse outcomes was analyzed.

**Results:**

A total of 312 patients with severe multiple injuries were included in this study, with an average age of 51.7 and a median Injury Severity Score (ISS) of 22.5. Among them, 115 patients developed coagulopathy, 169 patients developed SIRS, 26 patients developed AKI, and 11 patients died during hospitalization. We found that Myoglobin (*r* = 0.225, *P* < 0.001), CK (*r* = 0.204, *P* < 0.001), LDH (*r* = 0.175, *P* = 0.002) were positively correlated with ISS. Myoglobin is an independent risk factor for coagulopathy (OR = 1.90, 95%CI: 1.45–2.49), SIRS (OR = 1.41, 95%CI: 1.10–1.79), and AKI (OR = 4.17, 95%CI: 2.19–7.95). CK is an independent risk factor for coagulopathy (OR = 1.30, 95%CI: 1.00-1.67), while LDH is an independent risk factor for SIRS (OR = 1.49, 95%CI: 1.17–1.89) and AKI (OR = 2.30, 95%CI: 1.43–3.69). Especially for AKI, Myoglobin had a good predictive effect (AUC = 0.804, 95%CI:0.716–0.891). The best cut-off value is when the Myoglobin value is 931.11 µg/L, at which point the sensitivity is 61.53% and the specificity is 87.41%.

**Conclusions:**

The admission muscle injury index can predict trauma complications such as AKI, early coagulation disease, and SIRS, especially AKI. Compared to CK and LDH, admission myoglobin can predict complications remarkably, even better than ISS, especially AKI. Routine testing of muscle injury indicators upon admission is meaningful and can help physicians identify and prevent the occurrence of complications.

## Introduction

Severe multiple injuries are not only direct damage to organs and tissues, but also important life-threatening factors due to post-traumatic complications that occur during the course of the disease. Patients with severe trauma often experience coagulation dysfunction, with 25% of trauma patients experiencing coagulation dysfunction immediately upon admission, resulting in a significantly higher mortality rate than patients with normal coagulation [1, 2]. Endothelial injury and systemic hypoperfusion are the initial causes of trauma-induced coagulopathy (TIC) [3]. Damaged tissue cells and activated neutrophils cause a significant release of DAMP. As a pro-inflammatory mediator, DAMP can activate white blood cells and complement systems, consume platelets, and the inflammatory response may be overactivated, excessively prolonged, and generalized due to severe tissue damage, leading to systemic inflammatory response syndrome (SIRS) [4–6]. The incidence of acute kidney injury(AKI) is very high after trauma, up to 50%, and leads to prolonged treatment and elevated mortality [7]. Multiple factors can lead to and exacerbate AKI after trauma, including posttraumatic hypovolemia, acute rhabdomyolysis, volume and nature of resuscitation fluid, and renal reperfusion induced by resuscitation [8]. Therefore, early identification and prediction of severe complications caused by trauma can help initiate preventive interventions to reduce mortality rates.

Myoglobin(Mb), creatine kinase(CK), and lactate dehydrogenase(LDH) are commonly used indicators of muscle injury, which can reflect the severity of muscle injury [9–11]. These three indicators of muscle injury may have a certain correlation with the occurrence of complications. Previous studies have shown that coagulation dysfunction and SIRS occur early after trauma, and can even occur upon admission [12]. There are research reports that trauma patients activate and gradually deplete the protein C system when acute traumatic coagulation disease occurs, followed by a clear tendency towards infection complications [13]. Muscle injury releases immune stimulating molecules, activates inflammatory cells and maintains an inflammatory state, and heme derived from myoglobin also promotes pro-inflammatory effects on endothelial and renal tubular epithelial cells, which further leads to the occurrence of AKI [14]. Many studies have reported the correlation between myoglobin and CK peaks and post-traumatic AKI [15–17]. In order to predict AKI as early as possible, some studies have also explored the predictive ability of admission myoglobin and CK for post-traumatic AKI [18, 19]. A study found through retrospective analysis of included articles that high levels of LDH can predict AKI [11]. Inflammation, coagulation and kidney injury are closely related and influenced each other, which constitute the process of the formation of severe post-traumatic complications [20]. Currently, there are few studies exploring the association between three muscle injury indicators and post-traumatic inflammation and coagulation, and comparing their predictive abilities.

In order to identify high-risk populations for trauma complications early on, implement clinical interventions as soon as possible, and reduce mortality rates, we conducted this study. Based on previous studies, we hypothesize that three admission muscle injury indicators (Mb, CK, LDH) can predict the occurrence of post-traumatic SIRS, coagulation disorders, and AKI. Based on this assumption, the aim of our study is to answer the following questions: (1) Is there a positive association between admission muscle injury indicators and injury severity? (2) Can admission myoglobin, CK, and LDH all predict post-traumatic complications well? (3)Which one or several is the best in predicting coagulation disorders, SIRS or AKI individually among these three biomarkers?

## Materials and methods

### Study design and participants

The retrospective study was conducted on patients who were admitted to the Trauma Center of China-Japan Union Hospital of Jilin University between July 2020 and October 2022. Upon admission, blood samples were collected from all patients. Laboratory biochemical tests were then performed, and patients’ basic information, clinical diagnosis, biochemical indicators, and survival status were subsequently recorded. The collected data was approved by the Ethics Committee of China-Japan Union Hospital of Jilin University (Ethical approval number:2023072601).

The in vitro quantitative determination of Mb, CK-MB, NT-proBNP in serum was performed by the immunoassay method based on chemiluminescence reaction and the automatic biochemical immune analyzer (VITROS 5600, Ortho Clinical Diagnostics, USA). The in vitro measurement of serum LDH and CK was performed using the dry chemical rate method (VITROS 5600, Ortho Clinical Diagnostics, USA). The in vitro quantitative measurement of serum creatinine was performed by Sarcosine oxidase method on ANLAU5800 automatic biochemical analyzer (Beckman Coulter, USA). TP, ALB and PALB are also measured by AU5800 automatic biochemical analyzer (TP adopts Biuret method, ALB adopts Bromocresol green method, and PALB adopts the immune turbidimetry method). APTT, PT, TT and FIB were measured by coagulation method in automatic hematology analyzer (BC-6900, China), D-dimer was detected by immunoturbidimetry, and WBC was detected by optical method (BC-6900, China).

**Fig. 1:**
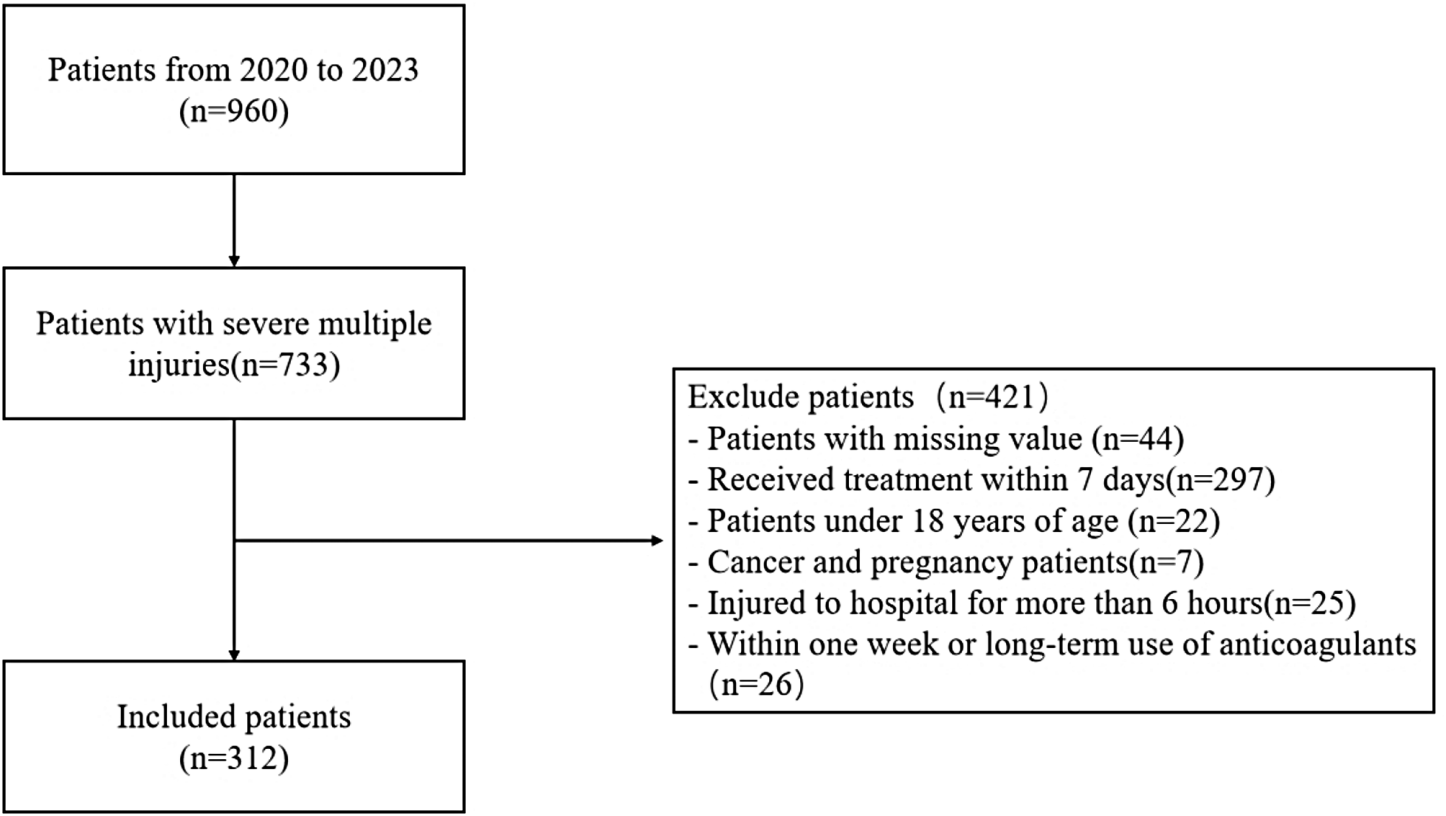
Flow chart of patients screening process, 312 patients with severe trauma were included

All patients were delivered to our hospital within 6 h. All patients included in this study were patients with severe multiple injuries whose ISS was greater than or equal to 16. All patient indicators were collected at the first time of admission, so none of the patients underwent drug therapy or infusion therapy. Because some of the patients had surgery at local hospitals, measures of population could interfere with the results, so people who had surgery within 7 days were also excluded. Patients younger than 18 years of age and those with pregnancy or cancer were excluded. Patients with missing clinical information were also excluded from the study, and the specific screening process is shown in the Fig. 1. In this study, In order to more intuitively see the relationship between indicators and ISS, patients were divided into three groups (16 < ISS ≤ 25, 25 < ISS ≤ 35, ISS > 35) based on the ISS severity grade and previous study grouping [21, 22]. We collected patients’ basic demographic information, complete blood count information, ion indicators, liver and renal function indicators, coagulation indicators, chest pain indicators, muscle injury indicators and corresponding clinical outcomes. Nonspecific SIRS criteria such as pyrexia or neutrophilia will continue to aid in the general diagnosis of infection [23]. SIRS is considered to be present when patients have more than one of the following clinical findings: Body temperature higher than 38 °C or lower than 36 °C, heart rate higher than 90/min, hyperventilation evidenced by respiratory rate higher than 20/min or PaCO2 lower than 32 mmHg, white blood cell count higher than 12,000 cells/ µl or lower than 4,000/ µl [24]. Patients with PT > 18s, INR > 1.5, APTT > 60s, or TT > 15s were diagnosed as coagulopathy patients [25]. Due to the lack of previous renal function information, 75 ml/min/1.73m^2^ was used as the estimated GFR [26, 27]. The creatinine level was calculated by referring to the modified MDRD(Modification of Diet in Renal Disease) formula in China [28, 29]. We used the KDIGO diagnostic criteria to determine AKI [30]. Serum creatinine (SCr) levels increased by more than 0.3 mg/dl (or 26.5 µmol/l) within 48 h, or patients with an increase in SCr to ≥ 1.5 times baseline within 7 days were diagnosed with AKI [31].

### Statistical analysis

In this study, normal continuous variables were described by means and standard deviations, while non-normal data were described by median combined IQRs. Categorical variables were described using frequency or percentage and chi-square tests were performed. The values of Myoglobin, CK, LDH were all log-transformed to satisfy the normal distribution.

We divided patients into three groups based on ISS. One-Way ANOVA test was used to compare the difference in indicators levels among all groups, and spearmen rank correlation was used to analyze the correlation between muscle injury indicators and ISS. Univariate and multivariate logistic regression were used to analyze the relationship between research variables and study outcomes, and muscle injury indicators were standardized to analyze the effect of each standard deviation change. Due to the limited retrospective data in this study, only age, gender, and ISS were selected as confounding factors for regression adjustment in this study. Finally, the clinical predictive value of each muscle injury indicators was indicated by the receiver operating characteristic curves. All statistical analyses were performed through SPSS24.0, and *P* < 0.05 was considered to be statistically significant.

## Results

### Baseline data for trauma patients with and without complications

This study included 312 patients with severe multiple injuries, of whom 36.86% developed traumatic coagulopathy, 54.17% developed SIRS, 8.33% developed AKI, and 3.53% died during hospitalization at the trauma center. The patients who participated in this study had a mean age of 51.7 ± 13.51 years, with 209 males accounting for 67.0% of all patients. The median ISS was 22.50 (18.00–27.00), and the median length of hospital stay was 15.00 (8.00–21.00) days. After calculation, the statistical power of statistical analysis between patients with coagulopathy, SIRS, AKI and those without complications was greater than 0.8 in this study.

Tables 1, 2 and 3 present the basic characteristics of the study population, stratified by coagulopathy, SIRS, and AKI, respectively, and we compared the differences in indicators between patients. The basic information and comparison of patients divided by length of stay are shown in supplementary materials 1. We observed significant differences in the levels of Myoglobin, Creatine kinase-MB, Fibrinogen, RBC, Total Protein, Albumin between patients with coagulopathy and non-patients, and ISS and death outcomes were also different. There were significant differences in Myoglobin, LDH, NT-proBNP, D-Dimer, Fibrinogen, WBC, PLT, NEUT%, LYM%, Total Protein, Albumin, and Serum creatinine between patients with SIRS and non-patients. For AKI patients, differences were found in Myoglobin, LDH, Creatine kinase-MB, NT-proBNP, WBC and UREA, and the death outcome of the patients was different. Overall, we found differences in muscle injury indicators between patients with all three complications in severe trauma patients and those without complications.

**Table 1:** Demographic data, clinical features, and laboratory indicators between patients with coagulopathy and without coagulopathy

Items	Coagulopathy (*n* = 115)	No Coagulopathy (*n* = 197)	t/χ^2^/Z	*P*-Value
Age	51.43 ± 14.78	51.86 ± 12.74	0.27	0.790
Sex				
Male	70(60.9)	139(70.6)	3.08	0.080
Female	45(39.1)	58(29.4)		
Death				
Yes	9(7.8)	2(1.0)	9.90	0.002
No	106(92.2)	195(99.0)		
ISS	24.00[20.00, 29.00]	22.00[18.00, 26.00]	-1.97	0.048
Hospitalization time (d)	16.00[8.00, 22.00]	14.00[8.50, 21.00]	-1.03	0.301
Myoglobin*	2.63 ± 0.48	2.35 ± 0.49	-4.87	< 0.001
Creatine Kinase (CK)*	2.68 ± 0.40	2.89 ± 0.42	-1.83	0.068
Lactate dehydrogenase (LDH)*	2.60 ± 0.21	2.59 ± 0.20	-0.51	0.613
Creatine kinase-MB (CK-MB)*	1.08 ± 0.46	0.88 ± 0.47	-3.65	< 0.001
N-Terminal Pro-Brain Natriuretic Peptide (NT-proBNP)*	2.06 ± 0.45	2.05 ± 0.46	-0.17	0.866
PT (s)	12.40[11.70, 13.80]	11.80[1.15, 12.70]	-4.96	< 0.001
APTT (s)	27.30[25.40, 29.40]	27.50[25.60, 29.80]	-1.17	0.862
INR	1.10[1.04, 1.22]	1.04[0.99, 1.12]	-5.00	< 0.001
TT (s)	16.10[15.30, 17.20]	13.80[13.00,14.30]	-14.74	< 0.001
D-Dimer (µg/L)	19.91[6.61, 46.22]	7.56[3.06, 16.67]	-5.33	< 0.001
Fibrinogen (g/L)	2.12[1.59, 2.55]	2.98[2.50, 3.81]	-9.58	< 0.001
WBC ($\:\times\:{10}^{9}/\text{L}$)	12.89[9.86, 16.27]	12.14[9.88, 15.36]	-0.94	0.345
RBC ($\:\times\:{10}^{9}/\text{L}$)	3.58 ± 0.73	4.09 ± 4.94	5.58	< 0.001
PLT ($\:\times\:{10}^{9}/\text{L}$)	186.00[146.00, 223.00]	197.00[154.00, 228.50]	-1.67	0.096
NEUT%	89.60[84.90, 91.70]	88.80[83.30, 91.05]	-1.32	0.186
LYM%	5.80[4.10, 9.40]	6.50[4.30, 10.20]	-1.13	0.260
Total Protein (TP, g/L)	56.38[49.10, 63.85]	63.65[56.41, 68.39]	-4.89	< 0.001
Albumin (g/L)	34.85[28.97, 39.40]	38.08[33.43, 41.65]	-3.61	< 0.001
UREA (mmol/L)	6.00[5.00, 7.64]	5.95[4.91, 7.61]	-0.26	0.797
Serum creatinine*	1.80 ± 0.14	1.80 ± 0.17	-0.46	0.645

^*^ indicates that the data was log transformed

**Table 2:** Demographic data, clinical features, and laboratory indicators between patients with SIRS and without SIRS

Items	SIRS(*n* = 169)	No SIRS(*n* = 143)	t/χ^2^/Z	*P*-Value
Age	49.55 ± 14.09	54.24 ± 12.36	3.10	0.002
Sex				
Male	114(67.46)	95(66.43)	0.04	0.848
Female	55(32.54)	48(33.57)		
Death				
Yes	8(4.73)	3(2.10)	1.58	0.208
No	161(95.27)	140(97.90)		
ISS	51.00[41.00, 58.00]	22.00[18.00, 26.00]	-0.50	0.615
Hospitalization time (d)	14.00[9.00, 20.00]	15.00[8.00, 21.00]	-0.18	0.858
Myoglobin*	2.53 ± 0.49	2.36 ± 0.50	-2.99	0.003
Creatine Kinase (CK)*	2.64 ± 0.20	2.61 ± 0.42	-0.64	0.522
Lactate dehydrogenase (LDH)*	2.63 ± 0.20	2.55 ± 0.19	-3.60	< 0.001
Creatine kinase-MB (CK-MB)*	1.00 ± 0.47	0.90 ± 0.48	-1.94	0.054
N-Terminal Pro-Brain Natriuretic Peptide (NT-proBNP)*	2.00 ± 0.42	2.11 ± 0.49	2.09	0.037
PT (s)	12.00[11.25, 13.10]	12.00[11.30, 13.00]	-0.32	0.752
APTT (s)	27.50[25.35, 29.90]	27.30[25.60, 29.60]	-0.09	0.928
INR	1.06[0.99, 1.16]	1.06[1.00, 1.15]	-0.30	0.766
TT (s)	14.50[13.60, 15.75]	14.40[13.40, 15.40]	-1.02	0.310
D-Dimer (µg/L)	15.15[5.20, 30.97]	7.20[2.81, 18.90]	-3.60	< 0.001
Fibrinogen (g/L)	2.55[2.01, 3.13]	2.84[2.25, 3.63]	-2.53	0.011
WBC ($\:\times\:{10}^{9}/\text{L}$)	15.36[13.54, 18.75]	9.58[7.59, 10.71]	-15.04	< 0.001
RBC ($\:\times\:{10}^{9}/\text{L}$)	4.01 ± 0.82	3.78 ± 0.77	-0.57	0.566
PLT ($\:\times\:{10}^{9}/\text{L}$)	207.00[172.00, 245.00]	173.00[140.00, 212.00]	-4.65	< 0.001
NEUT%	90.00[87.45, 92.00]	85.60[77.50, 90.10]	-6.59	< 0.001
LYM%	5.10[3.60, 6.95]	8.20[5.70, 14.40]	-6.96	< 0.001
Total Protein (TP, g/L)	63.28[54.04, 68.39]	59.29[51.23, 66.04]	-2.34	0.019
Albumin (g/L)	38.36[31.51, 41.68]	35.46[30.40, 39.40]	-2.93	0.003
UREA (mmol/L)	5.92[4.93, 7.58]	6.22[4.99, 7.66]	-0.38	0.702
Serum creatinine*	1.82 ± 0.17	1.78 ± 0.15	-2.05	0.042

^*^ indicates that the data was log transformed

**Table 3:** Demographic data, clinical features, and laboratory indicators between patients with AKI and without AKI

Items	AKI(*n* = 26)	No AKI(*n* = 286)	t/χ^2^/Z	*P*-Value
Age	53.35 ± 17.91	51.55 ± 13.06	-0.65	0.518
Sex				
Male	20(76.92)	189(66.08)	1.27	0.260
Female	6(23.08)	97(33.92)		
Death				
Yes	5(19.23)	6(2.09)	15.84	< 0.001
No	21(80.77)	280(97.89)		
ISS	24.50[22.00, 29.00]	22.00[18.00, 26.25]	-1.69	0.090
Hospitalization time (d)	10.00[4.00, 21.50]	15.00[9.00, 21.00]	-1.50	0.134
Myoglobin*	2.93 ± 0.38	2.41 ± 0.49	-5.25	< 0.001
Creatine Kinase (CK)*	2.76 ± 0.39	2.61 ± 0.41	-1.76	0.080
Lactate dehydrogenase (LDH)*	2.73 ± 0.17	2.58 ± 0.20	-3.78	< 0.001
Creatine kinase-MB (CK-MB)*	1.16 ± 0.48	0.94 ± 0.47	-2.36	0.019
N-Terminal Pro-Brain Natriuretic Peptide (NT-proBNP)*	2.39 ± 0.60	2.02 ± 0.43	-4.00	< 0.001
PT (s)	12.45[11.08, 14.48]	12.00[11.30, 13.00]	-1.06	0.291
APTT (s)	28.15[24.65, 31.20]	27.35[25.58, 29.60]	-0.47	0.638
INR	1.11[0.98, 1.28]	1.06[1.00, 1.15]	-1.04	0.297
TT (s)	15.15[14.05, 18.08]	14.40[13.40, 15.40]	-2.66	0.008
D-Dimer (µg/L)	24.77[5.78, 78.80]	13.17[3.85, 22.62]	-2.61	0.009
Fibrinogen (g/L)	2.38[1.47, 3.08]	2.71[2.18, 3.36]	-1.85	0.064
WBC ($\:\times\:{10}^{9}/\text{L}$)	17.43[11.52, 19.48]	12.17[9.78, 15.36]	-2.98	0.003
RBC ($\:\times\:{10}^{9}/\text{L}$)	3.77 ± 0.87	3.91 ± 0.80	0.88	0.379
PLT ($\:\times\:{10}^{9}/\text{L}$)	184.50[129.00, 227.00]	192.00[152.75, 226.25]	-0.75	0.456
NEUT%	88.05[84.08, 92.43]	88.90[83.70, 91.43]	-0.04	0.971
LYM%	6.50[4.05, 10.18]	6.20[4.20, 9.75]	-0.01	0.995
Total Protein (TP, g/L)	55.96[49.59, 64.39]	61.93[53.57, 67.45]	-1.77	0.077
Albumin (g/L)	33.79[30.52, 40.55]	37.20[31.25, 40.46]	-1.13	0.257
UREA (mmol/L)	7.61[5.77, 10.34]	5.92[4.88, 7.45]	-3.62	< 0.001
Serum creatinine*	2.08 ± 0.31	1.77 ± 0.11	-11.15	< 0.001

^*^ indicates that the data was log transformed

### Relationship between muscle injury indicators and trauma severity

**Fig. 2:**
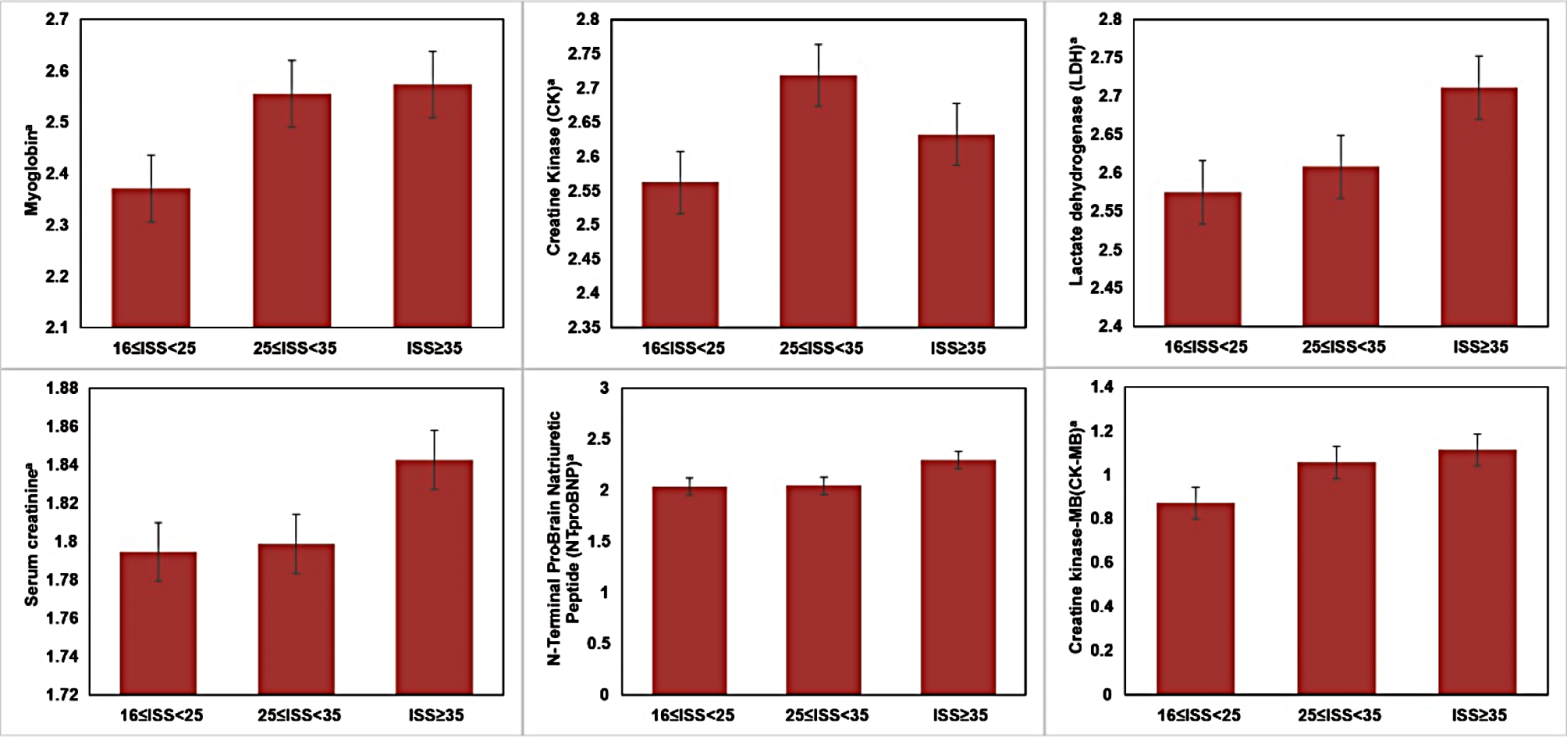
Distribution of trauma related indicators among different groups

Multiple groups of One-Way ANOVA test and linear trend test were performed to compare the differences among different trauma severity groups, and the results showed that Myoglobin, CK, LDH, Creatine kinase-MB levels were were significantly different (Fig. 2, Supplementary Table 2). Spearman correlation analysis showed that Myoglobin (*r* = 0.225, *P* < 0.001), CK (*r* = 0.204, *P* < 0.001), LDH (*r* = 0.175, *P* = 0.002), Creatine kinase-MB (*r* = 0.216, *P* < 0.001), and Hospitalization time (*r* = 0.167, *P* = 0.003) were positively associated with ISS, which means that there is a positive correlation between muscle injury indicators and trauma severity. The linear trend test also indicates that LDH may rises as ISS increases (Supplementary Table 2).

### Influence of muscle injury indicators on trauma complication

Through baseline data analysis, we found significant differences in muscle injury indicators between patients with coagulation disease, SIRS, and AKI and those without these complications. Then we used logistic regression to analyze the specific effects of various muscle injury indicators on different complications. In a crude analysis, we found that some muscle injury indicators were associated with the prevalence of coagulopathy. Among them, Myoglobin (OR = 1.82, 95%CI: 1.41–2.35) was a risk factor for coagulopathy. In the meantime, CK-MB (OR = 1.53, 95%CI: 1.20–1.97) also was a risk factor for the incidence of coagulopathy. And only after adjusting for confounding factors, CK (OR = 1.30, 95%CI: 1.00-1.67) was a risk factor for coagulopathy (Table 4). In the analysis of SIRS and AKI, Myoglobin (OR = 1.41, 95%CI: 1.10–1.79), LDH (OR = 1.49, 95%CI: 1.17–1.89) were risk factor for SIRS. In the crude analysis, NT-proBNP (OR = 0.79, 95%CI: 0.63–0.99) was protective factor for SIRS, however, after adjusting for confounders, NT-proBNP was not predictive of SIRS (Table 5). At the same time, Myoglobin (OR = 4.17, 95%CI: 2.19–7.95), LDH (OR = 2.30, 95%CI: 1.43–3.69), CK-MB (OR = 1.54, 95%CI: 1.01–2.36) and NT-proBNP (OR = 1.98, 95%CI: 1.36–2.89) were also risk factors for AKI. Moreover, SIRS (OR = 2.62, 95%CI: 1.05–6.52) was also found to be a risk factor for AKI (Table 6). With regard to death, we found that muscle injury indicators could not independently predict death outcomes, but coagulopathy (OR = 5.80, 95%CI: 1.15–29.41) and AKI (OR = 11.70, 95%CI: 2.44–56.02) were risk factors for death (supplementary Table 5).

**Table 4:** Simple and multiple logistic regression analyses for the association between laboratory test results with coagulopathy

Items	Unadjusted OR (95% CI)	*P*-Value	Adjusted OR (95% CI)^b^	*P*-Value
Myoglobin^a^	1.82(1.41, 2.35)	< 0.001	1.90(1.45, 2.49)	< 0.001
Creatine Kinase (CK)^a^	1.25(0.98, 1.58)	0.069	1.30(1.00, 1.67)	0.047
Lactate dehydrogenase (LDH)^a^	1.06(0.84, 1.34)	0.611	1.02(0.80, 1.30)	0.879
Creatine kinase-MB (CK-MB)^a^	1.54(1.21, 1.95)	< 0.001	1.53(1.20, 1.97)	0.001
N-Terminal Pro-Brain Natriuretic Peptide (NT-proBNP)^a^	1.02(0.81, 1.28)	0.866	0.98(0.76, 1.28)	0.906
Serum creatinine^a^	1.06(0.84, 1.32)	0.645	1.13(0.88, 1.46)	0.328

^a^ indicates that the data was divided by standard deviation to normalize

^b^ represents logistic adjustment for confounding factors age, gender, race and ISS

**Table 5:** Simple and multiple logistic regression analyses for the association between laboratory test i results with SIRS

Items	Unadjusted OR (95% CI)	*P*-Value	Adjusted OR (95% CI)^b^	*P*-Value
Myoglobin^a^	1.41(1.12, 1.77)	0.003	1.41(1.10, 1.79)	0.006
Creatine Kinase (CK)^a^	1.08(0.86, 1.34)	0.521	0.99(0.78, 1.26)	0.948
Lactate dehydrogenase (LDH)^a^	1.51(1.20, 1.91)	0.001	1.49(1.17, 1.89)	0.001
Creatine kinase-MB (CK-MB)^a^	1.25(1.00, 1.57)	0.054	1.22(0.96, 1.54)	0.100
N-Terminal Pro-Brain Natriuretic Peptide (NT-proBNP)^a^	0.79(0.63, 0.99)	0.039	0.88(0.68, 1.13)	0.322
Serum creatinine^a^	1.30(1.00, 1.68)	0.047	1.46(1.08, 1.98)	0.014

^a^ indicates that the data was divided by standard deviation to normalize

^b^ represents logistic adjustment for confounding factors age, gender, race and ISS

**Table 6:** Simple and multiple logistic regression analyses for the association between laboratory test results with AKI

Items	Unadjusted OR (95% CI)	*P*-Value	Adjusted OR (95% CI)^b^	*P*-Value
SIRS	2.46(1.00, 6.04)	0.049	2.62(1.05, 6.52)	0.040
Myoglobin^a^	4.34(2.31, 8.16)	< 0.001	4.17(2.19, 7.95)	< 0.001
Creatine Kinase (CK)^a^	1.51(0.95, 2.41)	0.083	1.45(0.89, 2.37)	0.135
Lactate dehydrogenase (LDH)^a^	2.29(1.45, 3.63)	< 0.001	2.30(1.43, 3.69)	0.001
Creatine kinase-MB (CK-MB)^a^	1.63(1.08, 2.47)	0.021	1.54(1.01, 2.36)	0.047
N-Terminal Pro-Brain Natriuretic Peptide (NT-proBNP)^a^	1.85(1.33, 2.57)	< 0.001	1.98(1.36, 2.89)	< 0.001
UREA	1.31(1.15, 1.50)	< 0.001	1.34(1.16, 1.56)	< 0.001

^a^ indicates that the data was divided by standard deviation to normalize

^b^ represents logistic adjustment for confounding factors age, gender, race and ISS

### Prediction effect of muscle injury indicators on trauma complications

**Fig. 3:**
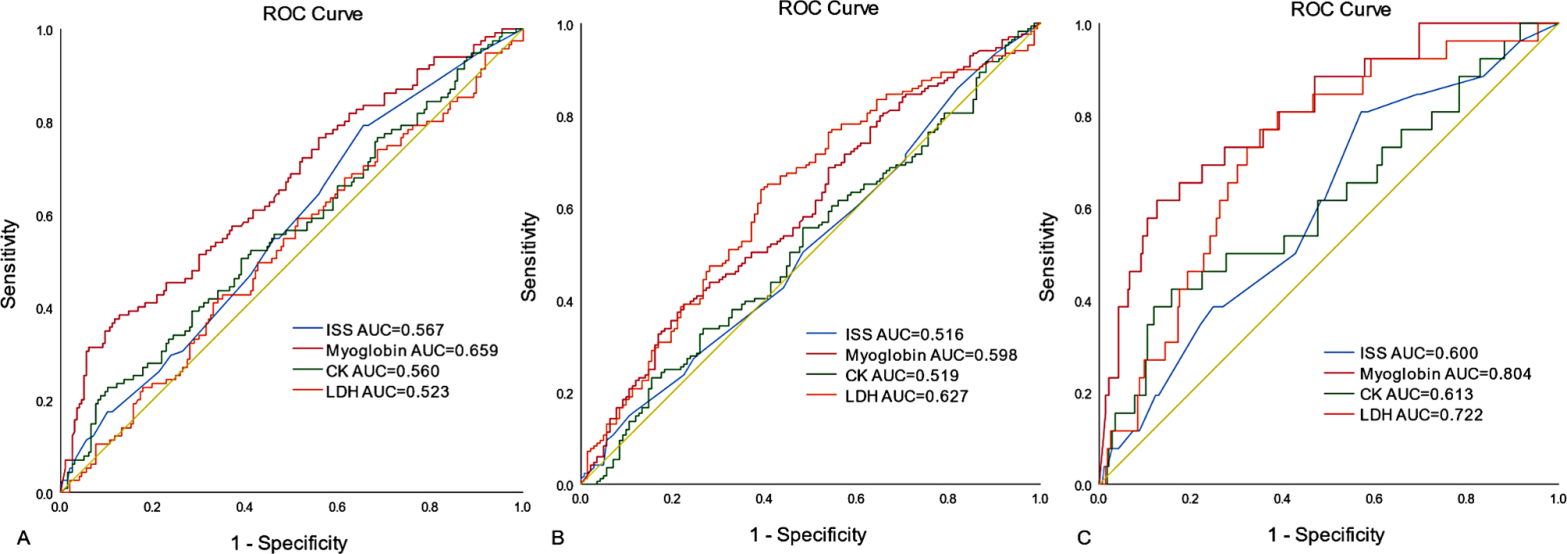
ROC curves of muscle injury indicators for predicting coagulopathy, SIRS, AKI

The clinical prediction effect of each muscle injury indicator on three trauma complications was analyzed, and the receiver operating characteristic curve was drawn. For coagulopathy and SIRS, the prediction effect of muscle injury indicators was limited, but the AUC value were all higher than ISS (Fig. 3A.B). For AKI, Myoglobin had a good predictive effect (AUC = 0.804, 95%CI:0.716–0.891), the prediction effects of other indicators were also better than ISS (Fig. 3C). The best cut-off value is when the Myoglobin value is 931.11 µg/L, at which point the sensitivity is 61.53% and the specificity is 87.41%.

## Discussion

We aimed to investigate whether there is an association between the severity and progression of multiple injuries and related indicators after muscle injury upon admission. We found, (1) there is an association between admission muscle injury indicators and the severity of multiple injuries. (2) The admission muscle injury indicators are related to the occurrence of multiple injury complications. (3) Myoglobin(Mb) is superior to creatine kinase(CK) and lactate dehydrogenase(LDH) in predicting complications of multiple injuries.

Previous studies have shown that physiological indicators such as GCS and systolic blood pressure are crucial in assessing the severity of injury in patients [32]. Compared to traditional anatomy based scoring systems, it can be obtained in a short period of time and represents the factors of body fluids and vital signs after injury, making up for the shortcomings of traditional scoring. The ISS is a commonly used method for assessing the severity of trauma internationally. ISS is not only a marker of tissue damage, but also closely related to mortality, which may ultimately caused by systemic inflammation, coagulation disorders and renal failure subsequently [5, 33].However, its ability to predict complications has significant limitations as it only represents the degree of damage to tissue structure, but not the organ function and possible complications.

Severe trauma patients inevitably experience varying degrees of muscle damage. Many studies have shown that myoglobin, CK, and LDH are important indicators for evaluating muscle injury [10, 11, 34]. This study used muscle injury indicators as physiological indicators and briefly explored the relationship between the level of muscle injury in the short term after injury and the severity of the injury. The level of muscle injury indicators at admission is related to ISS, but the association is weak in certain groups. The reason for this result is that in trauma patients with only or in combination with chest and(or) abdominal organ damage, the ISS score may increase, but at this time, the products released by skeletal muscles may not be significantly elevated. By comparing the indicators of different ISS groups, we found significant differences in Mb, CK, LDH, and CK-MB levels. Perhaps it is because as the levels of ISS between groups increase, the proportion and degree of severe muscle damage increase, and muscle cells are severely damaged, releasing more intracellular substances. This study confirms that these three muscle injury indicators are generally better than ISS in predicting complications in trauma patients admitted to hospital. The reason may be the subjective and delayed nature of ISS, and ISS only evaluates anatomical injuries, but cannot evaluate pathophysiological changes, which has certain deficiencies in assessing potential risks.

The recognition of TIC often relies on the extension of PT, APTT, and INR. However, the threshold for traditional coagulation test indicators is controversial [35]. Moreover, PT, APTT, and INR only reflect the contribution of plasma proteins to clot formation, ignoring the role of platelets. The addition of platelet count and Claus assay for measuring fibrinogen levels resulted in increased time to obtain results from multiple tests and the inability to identify excessive fibrinolysis [20]. At present, the best alternative is Viscoelastic hemostasis assay(VHA), which can provide multiple measurement values, real-time data, and quickly obtain results. The biggest advantage is that it can identify severe fibrinolysis [20, 36, 37]. But the high cost brought by VHA is difficult to popularize in resource poor areas. Moreover, TIC is a complex pathophysiological process that involves endothelial damage, platelet changes, coagulation factor consumption, inflammation, and immune dysfunction [38]. At present, there is no single detection method that can integrate the changes of the above factors and provide a comprehensive evaluation for clinical applications [20, 39].

In our study, elevated myoglobin levels after tissue injury were found to be a risk factor for the occurrence of traumatic coagulation disease. Compared to CK, it can better predict the occurrence of coagulation disease. The main reason for this result is that, in addition to myoglobin and CK reflecting to some extent the degree of muscle cell and tissue damage, the upregulation of heme derived from myoglobin α Chemokines and interleukin-8 (IL-8) genes are expressed, and neutrophils and protein kinase C systems are activated [14, 40]. The activation of the protein kinase C system is one of the main mechanisms of TIC, which lead to the cleavage of FVa and FVIIIa, as well as the de inhibition of fibrinolysis, achieving a dual anticoagulant effect [41, 42].

Post traumatic inflammatory response can be overactivated, excessively prolonged, and generalized due to severe tissue damage [5].There is research indicating a strong correlation between enhanced neutrophil response and muscle protein release. Compared to creatine kinase, myoglobin is more likely to leak from damaged muscles and disappear from circulation faster due to renal excretion, leading to a more acute inflammatory response [43].LDH is currently a commonly used inflammatory marker, and the explanation for the predictive ability of myoglobin is that it reflects the degree of tissue damage and renal function impairment. In fact, the number of infiltrating macrophages and levels of pro-inflammatory cytokines are positively correlated with renal dysfunction and tissue damage [44].In this study, admission myoglobin and LDH levels were associated with the progression of SIRS, but their predictive ability was limited, with LDH slightly better than myoglobin.

The vast majority of research on muscle injury indicators is limited to myoglobin and CK, and is based on rhabdomyolysis, exploring their effects on rhabdomyolysis and AKI [19, 45]. Some studies focus on the association between myoglobin peaks, CK peaks, rhabdomyolysis, and AKI [15, 19, 46]. The deposition of myoglobin causes renal vasoconstriction and exacerbates renal damage due to its direct toxic effect on renal tubules [47]. CK itself does not have nephrotoxicity and is not a direct mediator involved in causing AKI, but its level is an indicator of the severity of intramuscular content release [46]. When CK does not reach its peak, it is not possible to accurately indicate the severity of cell content release with nephrotoxicity, and thus cannot predict AKI. The time for CK to reach its peak is 17 h. Although myoglobin reaches its peak earlier than CK, both peaks are in the late stage of rhabdomyolysis and cannot be used as early predictive indicators of AKI [15, 17, 19, 46]. To address this issue in trauma patients, our studies have used myoglobin, CK and LDH measured immediately upon admission. Many studies have shown a significant increase in LDH concentrations in AKI and rhabdomyolysis [11, 48, 49]. However, the ability of LDH to predict AKI induced by rhabdomyolysis has not been reported. This study confirms the predictive ability of admission LDH for AKI at the onset of rhabdomyolysis after muscle injury. This result may be related to the progression of post-traumatic inflammatory response, and the specific mechanism is not clear. However, due to the distribution of LDH in all tissues and cells throughout the body, an increase in LDH can indicate damage to multiple tissues with poor specificity, which may be used as an auxiliary diagnosis for muscle injury induced AKI only.

In order to evaluate the ability of various muscle injury indicators to predict AKI, this study grouped admission LDH with previous studies on admission myoglobin and CK and compared them. It was found that myoglobin performed the best in predicting AKI, followed by LDH, and CK could not be predicted. This is related to the mechanism of AKI caused by rhabdomyolysis, where myoglobin is a key driving factor.

Our research results indicate that admission muscle injury indicators can predict the occurrence of traumatic complications. Among them, myoglobin has the best predictive ability, especially for AKI. The admission CK and LDH level can serve as an auxiliary reference in predicting AKI. The three measures of admitted muscle injury were slightly less effective in predicting TIC and SIRS than in predicting AKI. The level of CK admitted to hospital in this study did not predict AKI ideally, which is inconsistent with the results of some studies, but it further confirms that myoglobin at admission is superior to CK in prediction. This study also provides certain clinical value, indicating that incorporating muscle injury indicators into routine screening of trauma patients upon admission is meaningful, particularly for patients with large muscle group injury, and has reference value for predicting trauma related complications, especially the occurrence of AKI.

In addition to Mb, CK, and LDH, this study also included other laboratory indicators to gain a more comprehensive understanding of the factors influencing the prognosis of trauma patients. CK-MB is an isoenzyme of CK, which is mainly distributed in cardiac muscle and also distributed in skeletal muscle. Like cardiac troponin, CK-MB is a routine laboratory test for AMI diagnosis. This study confirmed that CK-MB is elevated in early coagulation disorders after skeletal muscle injury, which may be because it was excessively released into the blood and detected after traumatic hemorrhagic shock. Also, we observed that NT proBNP is a risk factor for AKI, and there is a significant difference between the AKI and non-AKI groups. NT proBNP has been extensively studied in heart failure and COPD, and is associated with hypoxia, inflammation, and cardiovascular stress [50]. We speculate that there is a correlation between post-traumatic stress status and NT proBNP. The increase in hormones (such as adrenaline and cortisol) caused by stress will enhance the traction of the ventricular wall for a period of time, triggering the release of NT proBNP [51]. In addition, tissue hypoxia caused by blood loss has been identified as a potential factor for the upregulation of NT proBNP levels [52, 53]. This systemic stress response simultaneously causes renal vasoconstriction, decreased renal blood flow, and decreased glomerular filtration rate, leading to secondary AKI. CK-MB and NT proBNP may be used as predictors of potential severe post-traumatic complications and will be promoted in our follow-up study.

It should be explained that the reason for the low mortality rate of severe multiple injuries in this study. Due to short-term transportation and the development of routine and effective treatment methods, the annual mortality rate has been consistently below 5% in this center.

We admit that there are certain limitations in this study. First, this study is a single-center retrospective data analysis with a small sample size. Second, this study lacks analysis of the dynamic changes in various indicators and the impact of injury sites after admission. Third, the lack of information on the patient’s use of nephrotoxic drugs and past renal function can have a certain impact on the research results. Fourth, evaluating AKI solely based on creatinine without considering urine output may underestimate the incidence of AKI. Fifth, treatments and techniques after admission of different patients can have an impact on the occurrence of complications by different supervising physicians. However, all physiological and biochemical indicators in this study were collected at the first time of admission, data with results exceeding the upper limit of the instrument may be subject to error and were not included in this study, so all data were accurate and reliable. In addition, our study is applicable to a wide range of severe trauma patients and has not been treated based on diagnostic criteria or other special screening methods for muscle injury.

## Conclusion

We fully illustrated that admission muscle injury indicators were risk factors for patients with severe trauma, demonstrated that they were positively associated with trauma severity, and showed their predictive effect on the occurrence of early coagulopathy, SIRS and AKI in trauma patients, and had a better significance than ISS. Notably, Myoglobin is an independent risk factor for early coagulopathy, SIRS and AKI in patients with multiple injuries. Therefore, the rapid detection of serum muscle injury indicators upon admission can assist physicians in assessing the severity of a patient’s injury and identifying potential complications.

## Data Availability

No datasets were generated or analysed during the current study.

## Abbreviations

APTT: Activated partial thromboplastin time
AKI: Acute kidney injury
ALB: Albumin
CK-MB: Creatine kinase-MB
CK: Creatine kinase
GFR: Glomerular filtration rate
LDH: Lactate dehydrogenase
LYM: Lymphocyte
INR: International normalized ratio
ISS: Injury severity score
KDIGO: Kidney disease improving global outcome
MDRD: Modification of diet in renal disease
NEUT: Neutrophils
NT-proBNP: N-terminal Pro-brain Natriuretic Pept
PT: Prothrombin time
PLT: Platelet
PALB: Prealbumin
RBC: Red blood cell
ROC: Receiver operating characteristics
SIRS: Systemic inflammatory response syndrome
SCr: Serum creatinine
TT: Thrombin time
TP: Total Protein
WBC: White blood cell
