# Correction: Role of the admission muscle injury indicators in early coagulopathy, inflammation and acute kidney injury in patients with severe multiple injuries

**DOI:** 10.1186/s13017-025-00638-y

**Published:** 2025-08-06

**Authors:** Liuquan Mu, Haideng Song, Mengdi Jin, Kaige Li, Yushan Guo, Nan Jiang

**Affiliations:** 1 Department of Trauma Center, China-Japan Union Hospital of Jilin University, Changchun, 130033 China; 2 Department of Emergency, Cheeloo College of Medicine, Weihai Municipal Hospital, Shandong University, Weihai, 264200 China; 3 Department of Epidemiology and Biostatistics, School of Public Health, Jilin University, Changchun, 130021 China

**Correction: World Journal of Emergency Surgery (2025) 20:19**

10.1186/s13017-025-00593-8

In this article [1], Mengdi Jin was mistakenly listed as a co-first author.

The original article has been corrected.
